# Supplementary material for: Words matter: political and gender analysis of speeches made by heads of government during the COVID-19 pandemic
Source: BMJ Glob Health. 2021 Jan 29;6(1):e003910. doi: 10.1136/bmjgh-2020-003910 (PMC7849321; doi:10.1136/bmjgh-2020-003910)
Supplement: Supplementary data [file bmjgh-2020-003910supp001.pdf]

## Appendix Table 1: Inclusion/Exclusion of countries

| *COUNTRY* | Leader | M/W | inclusion Rationale | Region | reason for exclusion |
|---|---|---|---|---|---|
| ***Belgium*** | Wilmès | W | G10, UNSC, Woman | Europe | Included |
| ***France*** | Macron | M | G10, UNSC | Europe | Included |
| ***Germany*** | Merkel | W | G10, UNSC, Woman | Europe | Included |
| ***United Kingdom*** | Johnson | M | G10, UNSC | Europe | Included |
| ***United States*** | Trump | M | G10, UNSC | North America | Included |
| ***Brazil*** | Bolsonaro | M | BRICS | South America | Included |
| ***Russia*** | Putin | M | BRICS, UNSC | Europe/Asia | Included |
| ***India*** | Modi | M | BRICS | Asia | Included |
| ***South Africa*** | Ramaphosa | M | BRICS, UNSC | Africa | Included |
| ***Dominican Republic*** | Medina | M | UNSC | Caribbean | Included |
| ***Indonesia*** | Widodo | M | UNSC | Asia | Included |
| ***Niger*** | Issoufou | M | UNSC | Africa | Included |
| ***Saint Vincent and the Grenadines*** | Gonsalves | M | UNSC | Caribbean | Excluded – Speeches not available on public domain |
| ***Tunisia*** | Fakhfakh | M | UNSC | Africa | Excluded – Speech transcripts not available, no capacity to transcribe Arabic |
| ***Vietnam*** | Nguyễn Phú Trọng | M | UNSC | Asia | Excluded – Speech transcripts not available, no capacity to transcribe Vietnamese |
| ***Bangladesh*** | Hasina | W | Woman | Asia | Included |
| ***Barbados*** | Mottley | W | Woman | Caribbean | Excluded – Only 1 speech available, other carribean nation chosen |
| ***Bolivia*** | Añez | W | Woman | South America | Included |
| ***Denmark*** | Frederiksen | W | Woman | Europe | Excluded – Only 1 speech available, other European countries already represented |
| ***Finland*** | Marin | W | Woman | Europe | Included |
| ***Iceland*** | Jakobsdottir | W | Woman | Europe | Excluded – Speeches not available on public domain |
| ***Namibia*** | Kuugongelwa | W | Woman | Africa | Excluded – Speeches not available on public domain |

| | | | | | |
|---|---|---|---|---|---|
| ***New Zealand*** | Ardern | W | Woman | Oceania | Included |
| ***Norway*** | Solberg | W | Woman | Europe | Included |
| ***Scotland*** | Sturgeon | W | Woman | Europe | Included |
| ***Serbia*** | Brnabic | W | Woman | Europe | Speeches not available on public domain |
| ***Sint Maarten*** | Jacobs | W | Woman | Caribbean | Included |
| ***Taiwan*** | Ing-wen | W | Woman | Asia | Included |

**Appendix 2: Sources for public statements/speeches**

জাতির উদ্দেশ্যে ভাষণ [Bangladesh]: প্রধানমন্ত্রীর কার্যালয়
গণপ্রজাতন্ত্রী বাংলাদেশ সরকার
প্রধানমন্ত্রীর কার্যালয় সংক্রান্ত; 2020 [Available from: https://pmo.gov.bd/site/view/pm-speech/%E0%A6%9C%E0%A6%BE%E0%A6%A4%E0%A6%BF%E0%A6%B0-%E0%A6%89%E0%A6%A6%E0%A7%8D%E0%A6%A6%E0%A7%87%E0%A6%B6%E0%A7%8D%E0%A6%AF%E0%A7%87-%E0%A6%AD%E0%A6%BE%E0%A6%B7%E0%A6%A3.

Sophie Wilmés News [Belgium]: Belgium Premier; 2020 [Available from: https://www.premier.be/en/timeline.

Jeanine Añez President of Bolivia [Bolivia] 2020 [Available from: https://www.youtube.com/channel/UC7QjQXQopwIvJ9KfmYj7EAQ.

Últimos Pronunciamentos [Brazil]: Presidência da República; 2020 [Available from: https://www.gov.br/planalto/pt-br/acompanhe-o-planalto/pronunciamentos.

Ante el CoronaVirus Danilo Medina habla al pueblo Dominicano [Dominican Republic]: Gobierno Danilo Medina; 2020 [updated 25 March 2020. Available from: https://www.youtube.com/watch?v=-h3vYohxADw.

Discurso presidente Medina por covid-19 [Dominican Republic]: Noticias SIN; 2020 [updated 17 March 2020. Available from: https://www.youtube.com/watch?v=rk63OZw8VHE.

Speeches [Finland]: Finnish Government; 2020 [Available from: https://valtioneuvosto.fi/en/current-issues/speeches.

Official speeches and statements 2020 [France]: Embassy of France in the United States; 2020 [Available from: https://franceintheus.org/spip.php?rubrique501.

Live from the Chancellery [Germany]: Die Bundeskanzlerin; 2020 [Available from: https://www.bundeskanzlerin.de/bkin-de/mediathek/live-aus-dem-kanzleramt.

Text Speeches [India]: Narendra Modi; 2020 [Available from: https://www.narendramodi.in/category/text-speeches.

Speech Transcripts [Indonesia]: Cabinet Secretariat of the Republic of Indonesia; 2020 [Available from: https://setkab.go.id/en/category/speech-transcript/page/4/.

Rt Hon Jacinda Ardern [New Zealand]: The official website of the New Zealand Government; 2020 [Available from: https://www.beehive.govt.nz/minister/rt-hon-jacinda-ardern.

Speeches [Niger]: Présidence de la République du Niger; 2020 [Available from: https://www.presidence.ne/discours-du-prsident.

Finn aktuelt [Norway]: Regjeringen; 2020 [Available from: https://www.regjeringen.no/no/aktuelt/aktuelle_saker/id2000005/?ownerid=&documenttype=aktuelt/talerogartikler&term=&page=4.

Transcripts [Russia]: President of Russia; 2020 [Available from: http://en.kremlin.ru/events/president/transcripts/page/4.

First Minister’s Speeches [Scotland]: Scottish Government; 2020 [Available from: https://www.gov.scot/collections/first-ministers-speeches/#2020.

Speeches [South Africa]: South African Government; 2020 [Available from: https://www.gov.za/speeches.

Government of Sint Maarten [Sint Maarten] 2020 [Available from: https://www.youtube.com/channel/UC1bIsTYvgBE1AZ75j9HlSBA.

News & activities [Taiwan]: Office of the President Republic of China (Taiwan); 2020 [Available from: https://english.president.gov.tw/Page/35.

News and communications, Prime Minister’s Office, 10 Downing Street [United Kingdom]: United Kingdom Government; 2020 [Available from: https://www.gov.uk/search/all?parent=prime-ministers-office-10-downing-street&content_purpose_supergroup%5B%5D=news_and_communications&organisations%5B%5D=prime-ministers-office-10-downing-street&order=updated-newest.

Briefings & Statements [United States]: White House; 2020 [Available from: https://www.whitehouse.gov/briefings-statements/?issue_filter=healthcare.

BMJ Publishing Group Limited (BMJ) disclaims all liability and responsibility arising from any reliance placed on this supplemental material which has been supplied by the author(s)
